# Agent-based models of the cultural evolution of occupational gender roles

**DOI:** 10.1098/rsos.221346

**Published:** 2023-06-28

**Authors:** C. P. Cross, L. G. Boothroyd, C. A. Jefferson

**Affiliations:** ^1^ School of Psychology & Neuroscience, University of St Andrews, St Mary's College, St Andrew, Fife KY16 9AZ, UK; ^2^ Department of Psychology, Durham University, South Road, Durham DH1 3LE, UK; ^3^ School of Computer Science, University of St Andrews, Jack Cole Building, North Haugh, St Andrews, Fife KY16 9SX, UK

**Keywords:** gender, gender roles, segregation, stereotypes, models, social learning

## Abstract

The causes of sex differences in human behaviour are contested, with ‘evolutionary’ and ‘social’ explanations often being pitted against each other in the literature. Recent work showing positive correlations between indices of gender equality and the size of sex differences in behaviour has been argued to show support for ‘evolutionary’ over ‘social’ approaches. This argument, however, neglects the potential for social learning to generate arbitrary gender segregation. In the current paper we simulate, using agent-based models, a population where agents exist as one of two ‘types’ and can use social information about which types of agents are performing which ‘roles’ within their environment. We find that agents self-segregate into different roles even where real differences in performance do not exist, if there is a common belief (modelled as priors) that group differences may exist in ‘innate’ competence. Facilitating role changes such that agents should move without cost to the predicted highest-rewards for their skills (i.e. fluidity of the labour market) reduced segregation, while forcing extended exploration of different roles eradicated gender segregation. These models are interpreted in terms of bio-cultural evolution, and the impact of social learning on the expression of gender roles.

## 1. Introduction

In industrialized societies, even those considered to be most gender egalitarian, women are overrepresented in some parts of the workforce and underrepresented in others [1]. Recent papers have used nation-level metrics to argue that gender differences in a range of personality traits and job-related preferences (such as STEM participation) correlate positively with measures of gender equality [2]. Some authors suggest that where women have social and political equality with men, they are free to choose career paths in line with their evolved predispositions [3]. For instance, Giolla & Kajonius [4] argue that ‘as gender equality increases both men and women gravitate towards their traditional gender roles'. This argument is in direct opposition to the ‘bio-social construction’ or ‘social role theory’ perspective, which predicts that as a society approaches gender equality, gender differences in psychological traits should decline [5]. In the current paper, we will argue that gendered social roles are best understood as products of domain-general social learning biases which served, in small-scale ancestral human societies, to guide individuals towards locally appropriate behaviour. We will argue that these formerly adaptive biases are currently operating in evolutionarily novel contexts, creating arbitrarily gendered patterns which will persist indefinitely in the absence of concerted efforts to oppose them.

Social learning allows animals of many species to acquire adaptive and often environmentally specific behaviours, such as complicated extractive foraging techniques, from conspecifics [6]. Learning has a clear adaptive benefit over reliance on narrow stereotypic behavioural repertoires [7], and learning socially avoids some of the risk and energetic costs of individual learning [8]. Copying is not used indiscriminately, however. Strategies used for guiding social learning in non-human species include: copying dominant or knowledgeable individuals [9]; copying behaviours which appear efficient [10]; or copying individuals of a particular sex (e.g. females among female-philopatric species, which is adaptive because of their greater experience in the local environment [10]). We propose here that the social learning strategies which begin to produce gendered behaviour in humans might be similar to those used by other species.

Like other animals, humans use social learning strategically [11,12]. Between the ages of 3 and 7 years, children acquire a tendency to prefer copying when making decisions alone is difficult [13]. Furthermore, children acquire tendencies to copy self-similar others in the first two years of life, whether the self-similarity takes the form of speaking the same language, general familiarity, or being of the same sex [14]. In societies with a gendered division of labour, any social learning bias that causes girls and boys to preferentially copy same-sex models would serve an adaptive function by equipping them with the skills they would most likely need in adulthood. Furthermore, sex-typing of behaviour can in some cases be produced by frequency-dependent copying in the absence of any sex bias. It has also been argued that humans may copy the behaviour of a majority because this is likely to be adaptive [7]. To the extent that a social group is segregated by gender, any tendency for children to adopt the most frequent behaviours of those around them will (after weaning) lead to their copying the behaviour of same-sex others. Whether children's social learning is sex-typed, or simply frequency-dependent, we argue that what children *perceive* to be the most common behaviours (in general, or of their own sex) will shape their behaviour—based on not just the nearby adults in their life but also those patterns of behaviour observed more widely, including through social institutions and other distal socialisation opportunities. Notably, social learning is not limited to overt behaviour, but includes strategies, information and attitudes. For example, Chinese children acquire gender-linked attitudes to mathematics representative of their peers' parents, predominantly via own-gender peers [15]. In this instance, peer-based social learning transmits (gendered) expectations regarding skills in a particular domain.

While testosterone might have some direct effects on behaviour via sex differences in infant temperament such as surgency (baseline energy and reactivity), evidence from industrialized populations also suggests an effect of testosterone on learning which facilitates the acquisition of locally sex-typed behaviour without dictating what those sex-typed behaviours are [5,16]. From infancy, children expect there to be categories of people (in general, not just regarding gender) which inform their inductive social reasoning [17]. This category formation precedes more complex processes such as stereotyping, requiring only that different categories are expected to differ in some way. Although children do not prefer to copy own-gender adults when presented with only one exemplar of each, when observing multiple male and female models making largely divergent choices, they are likely to copy the choices of their own gender [18,19]. However, Hines and colleagues have demonstrated that typically developing girls are more likely than girls with increased prenatal androgen exposure to copy female-typed behaviours in multiple-model learning paradigms [16,20]. This suggests that testosterone (or lack of) may prime learning of locally sex-typed behaviours, without making it necessarily more likely that any *specific* behaviour pattern (e.g. interest in things versus people) will be learned [21].

An important factor in the use of social learning is niche diversity: social learning is particularly powerful when there is a wide range of available behaviours to choose from and the adaptive landscape is relatively opaque [8]. As we have previously argued, gendered division of labour is likely adaptive in non-industrialized settings, but the specific skills required vary by ecology [22]. In industrialized societies, the range of available niches has expanded far beyond those in non-industrial subsistence societies, and physical constraints such as reproductive function, body size or strength are typically of little use in choosing between them. In this evolutionarily novel context, a bias that was initially adaptive in pre-industrial contexts (i.e. preferential copying of self-similar individuals) could now be driving arbitrary forms of gendered behaviour, such as the persistence of gender segregation found in workforces in industrialized societies. Mathematical modelling suggests that expansion of available niches drives a corresponding expansion of variability in personality traits, and empirical data show that personality is more variable in industrialized societies than in small-scale societies [23,24]. Where there is more variability in personality traits, there is more scope for men and women to differ in a manner which has been shaped by cultural learning. The dramatic shift in computing from being a female-dominated profession to a male-dominated one, for example, illustrates that the association of gender with behaviour is malleable and can be arbitrary [25].

A key point of the model we test below is thus the difference between non-industrial subsistence type populations and post-industrial populations with regard to sex differences. In subsistence societies, psychological and cognitive sex differences should reflect, more or less directly, the divergence of male and female roles within that society. For instance, behavioural segregation of children among foragers is related to the subsistence activities of their parents [26]. Similarly, while Vashro *et al*. found sex differences in spatial cognition among the Twe and Himba, where adult social roles include very divergent geographical mobility [27], Trumble *et al*. found no sex difference in spatial cognition among the Tsimane, where men and women tend to have similar geographical travel patterns [28]. As such, it seems that spatial cognition abilities are shaped by gender-specific social roles and routines in subsistence societies. Additionally, in any society, cultural products with which children engage in a gendered manner also shape skills and expectations of skill. For instance, giving women practice with first-person-shooter computer games has been shown to narrow sex differences in spatial tasks [29]. There is also evidence that early subsistence-linked sex roles may perpetuate via culture; Alesina *et al*. have argued that cultures which previously relied on the plough (requiring greater strength and ergo advantages for men in this role) show higher sex role differentiation than those which did not, even where farming has since mechanized [30]. Cultural processes thus have the potential to continue, and even magnify, differences seen in subsistence contexts.

We argue that sex differences in behaviour can emerge from a simple tendency to preferentially learn from self-similar others. This tendency will produce adaptive gendered behaviour in circumstances where there are actual sex differences in ability to perform tasks. However, because a tendency to copy self-similar others does not dictate which behaviours are to be learned, it will also produce gendered behaviour when there is no adaptive benefit. We tested these hypotheses in a series of agent-based models. Agent based modelling is a valuable technique which allows the exploration of both likely outcomes and boundaries on plausible developments over time of characteristics within a population [31]. We will show how actual sex differences in the ability to perform a role interact with cultural expectations of skill in a population of 1000 agents, to create gendered segregation in labour. We then remove sex differences in ability while leaving gendered learning intact and examine the conditions under which this does or does not reduce gender segregation in the labour force.

The aim of our models was to understand factors that cause agents to become more or less segregated by type. We model a population of 1000 agents of two different ‘types’, over 400 timesteps, corresponding to approximately 20 generations. Each agent tries out different roles in turn from a range of 60 options and, if the payoff is satisfactory, continues to perform it until either the payoff becomes unsatisfactory or the agent dies. Agents would explore new, unknown roles if their payoff from known roles dropped below: a) 100%, b) 95%, c) 90% or d) 75% of their satisfactory value (see Methods). This changing threshold mimics different levels of impedance causing people to remain in unsatisfactory jobs (e.g. the costs of retraining, etc).

We modelled four scenarios regarding the true relationship between agent type and ability to perform roles: In Model 1 (real large differences), substantial ability differences (Cohen's *d* = 1.5) exist between agent types for 40% of the roles. This is intermediate between the size differences that have been observed in armed assault between men and women [32] and interest in ‘boy's’ toys between girls and boys [33]. In Model 2 (real small differences) a smaller ability difference exists (*d* = 0.4), in line with typical differences between men and women on performance in a range of questionnaire and psycho-behavioural tests [34] for the same proportion of roles. In Model 3 (no differences), there are no ability differences according to agent type. Model 4 (vanishing differences) ran as Model 1 for 40 timesteps and then as Model 3 for the rest of the timesteps to mimic a situation where technology lessens the effect of size or strength on effective performance of tasks. We also varied how agents explored their world. Models 5 and 6 (extended exploration) are variants of Model 4 in which agents must spend their first 4 timesteps (Model 5; as if students were forced to complete 4 sequential apprenticeships across the college period) or 10 timesteps (Model 6; equivalent to roughly half a ‘working life’) trying different roles at every timepoint, to mimic a scenario in which individuals have a broad compulsory work experience curriculum into adulthood.

We also modelled five different patterns of globally held priors which modulated the degree of social learning agents engaged in: in prior condition 0, agents have a strong prior belief that agents are equal in ability and will consequently ignore gendered social information. In conditions 1–4, agents believe that there are: (1) few small differences, (2) few large differences, (3) many small differences, and (4) many large differences. These priors reflect agents' expectations regarding the utility of copying own-group patterns, versus (or in addition to) learning about their own performance in a given role. Thus, a set of priors that a small number of roles have a small group difference in average performance would represent a very low level of own-group copying bias. The priors for more, or stronger, group differences could be seen as representing stronger cultural expectations of gender differences and are effectively expressed as assigning a greater weight to social information when selecting roles. Thus, the more, or stronger, differences agents expect, the stronger their social learning (their copying bias). For comparison purposes, we also ran the models under conditions in which agents, rather than having priors, knew their skill levels in each role (perfect knowledge) and so could easily self-select into those roles which were more suited to them.

All together, these models allow us to tease apart the effects of belief in group differences in ability, from the existence of actual group differences, and show the extent to which the results differ from optimal adaptive behaviour.

## 2. Methods

### 2.1. Structure of model

We model a population of 1000 agents. Agents exist in two types which we arbitrarily denote ‘type 0’ and ‘type 1’. For simplicity, we assume that agents know which type they are and this does not change. Time passes in discrete steps, during which each agent acts in an order determined at random for that timestep. Each agent either dies and is replaced with a naive agent of the same type (5% chance), or performs one of 60 roles and earns a corresponding payoff. The payoff is determined by the agent's ability to generate goods in that role multiplied by the price of goods produced by that role in that timestep. The price of a good depends in turn on its scarcity, so agents are better off if they produce rare goods (in order to force division of labour across roles within the population). Agents search for a role to perform using three sources of information as follows.

### 2.2. Priors

The first source of agent information is a set of globally held priors which describe the probability that a previously unseen role will favour agents of type *0*, type *1*, or neither type. A prior has two important values. The first value is the proportion of roles that are expected to favour one type or the other, which can be: none favouring either type; 0.1 favouring each type (few differences); or 0.4 favouring each type (many differences). The second value is the size of any expected type difference in ability relative to neutral, which can be 0.2 (small) or 0.5 (large) of a standard deviation in overall agent ability (note because priors are coded as relative to neutral, by adding and subtracting that prior during calculations, the expected difference between types is twice that number). Agents use priors by modulating their predictions regarding which roles will favour them and by how much, when using social information (below). We model five values of agent priors: (i) belief in no differences for any role; (ii) belief in few small differences; (iii) belief in few large differences; (iv) belief in many small differences; (v) belief in many large differences. It should be noted that an agent with priors of type zero does not have ‘no prior beliefs’—they have a strong belief that the agents are equal in their ability and will, in effect, ignore gendered social information. In order to observe ‘optimal’ segregation, an additional ‘perfect knowledge’ condition was also created in which agents knew their own ability levels in all roles rather than estimating this using priors/observation/experience.

### 2.3. Social information

Agents also know the number of still-living agents of each type who have tried a role and either abandoned it or continued to perform it. From this (combined with publicly available knowledge of the value of goods produced by each role) the likelihoods that a role favours agents of type 0, type 1 or neither type, can be inferred (modulated by priors regarding size of difference between agents should a difference exist). These likelihoods are multiplied by the prior regarding proportion of roles that differ to generate an estimated probability that a role favours an agent's own type, and thereby estimate that agent's expected payoff from that role (factoring in again the prior for type difference in payoff). As such, if an agent has *any* prior that differences exist, then a bias in the numbers of agents in a role will lead to a higher predicted probability that role favours the more numerous agent type (and thus will give them a higher or lower payoff depending on their own type). Priors for larger differences or more differences will magnify that effect through the multiplications while calculating probability of the role favouring each type or none (the A0/A1/A2 calculations in the code). As noted above, if agents have an egalitarian prior, they ignore social information regarding agent type distribution. See electronic supplementary material, ODD Protocol, for detailed calculations.

### 2.4. Thresholds

Agents select the role with the highest expected payoff or, in the event of a tie, one of the top-ranked roles at random. When an agent performs a role once, they gain a third source of information, which is perfect knowledge of their ability to perform that role. This value is compared with that agent's threshold (which equals the estimated payoff of further search). If the value of an already-explored activity exceeds threshold, that activity becomes their occupation. They continue to perform the role until they die, unless the payoff drops below threshold because the price of the resulting good falls. They will then search for a new role as described above. We model the decision to switch between roles in three different ways: agents shift (a) in response to any drop below threshold, (b) if their payoff drops below 90% of threshold, (c) if their payoff drops below 75% of threshold.

### 2.5. Segregation index

The outcome of interest is the segregation index at timestep 400 (by which time the model is stable). The segregation index is the weighted mean of all roles' absolute deviation from a type ratio of 50 : 50, multiplied by 2. The minimum possible value is 0 (i.e. all roles have an evenly split number of type 0 and type 1) and the maximum value is 1 (i.e. all roles are performed by one agent type only). In practice, we rarely observe values below 0.2 because the mean number of agents per role is fewer than 20, such that a single agent above or below the expected count represents a substantial proportion for that role.

### 2.6. State of reality/type differences in ability

For each role, the true ability of each agent is drawn from a normal distribution with mean = 100 and standard deviation = 20. We then modify the true ability of agents in one of four ways. In Model 1, large differences exist: we create type differences in ability by adding 15 to ability scores for type 0 and subtracting 15 for type 1 in 20% of roles and vice versa for another 20%, resulting in an effective *d* score of 1.5. In Model 2, small differences exist: we modulate ability scores in an identical manner such that *d* = 0.4. In Model 3 no real differences between types exist, and we make no modifications to the abilities drawn from the normal distribution. In Models 4–6 agents have type differences as per Model 1 for the first 40 time steps but agents created after timestep 40 have no type differences.

### 2.7. Forced exploration

In Models 1–4 agents explore or exploit according to their individual assessments of their best option. In Models 5 and 6 agents must spend a period of time exploring different roles. We run the Model 4 state of reality (real differences for agents created in the first 40 time steps, but none thereafter) but add a constraint so that each agent must try at least 4 (Model 5) or 10 (Model 6) roles before choosing an occupation.

### 2.8. Final output

Because the models are stochastic, we ran each Model type/priors/inertia threshold combination 100 times and recorded the level of segregation within the population at each time point. The model is further elaborated in the electronic supplementary material, ODD Protocol document, and model code can be found at https://github.com/ChrisJefferson/sexdiffpub or archived on Zenodo [35]. Model output data and visualization/statistical analysis files are included as electronic supplementary material.

## 3. Results

In order to establish the ‘optimal’ outcome for agents under each state of reality average segregation was calculated to the final timepoint of each model type under the ‘perfect knowledge’ agent condition. This suggested that where large differences existed, agents ‘should’ end up distributed such that the segregation index was 0.51 (which would arise if all roles have roughly 75 : 25 splits, or half of roles are 100% segregated). Where small differences existed the optimal outcome was a 0.28 segregation index. For all models where type differences did not exist (Model 3) or ceased to exist (Models 4–6), optimal segregation was 0.19, likely due to random ability distributions and limits on the numbers of agents in any given role not always allowing perfect 50:50 splits.

**Figure 1.**
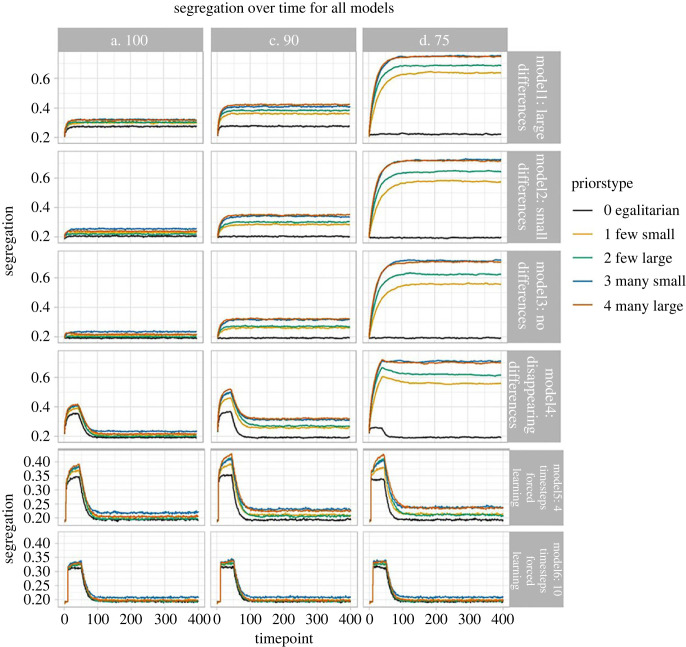
Model outcomes for each type of model and set of priors under 100%, 90% and 75% threshold for moving jobs (Agent remains in current job if it pays 100/90/75% or more of alternative). (Note scale of axis differs for Models 5 and 6). Figure created using ggplot2 [36].

**Figure 2.**
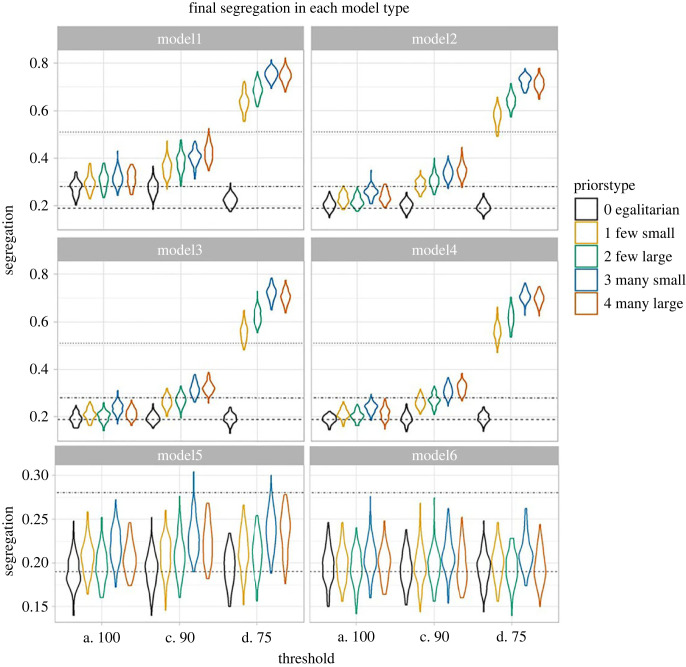
Final segregation distributions for each model type. (Note models 5 & 6 presented on different *y* axis scale). Dotted lines show approximate level of segregation observed when agents have perfect knowledge of skill levels under Model 1 conditions (top dotted line), Model 2 (conditions (middle dot-dash line) and Model 3 conditions (lower dashed line). Note only the lower two lines are shown for models 5 and 6.

Average segregation at each time point in each model is shown in figure 1; figure 2 shows distribution of final segregation levels in each model. Visual inspection of the plots shows that priors always increased the final level of segregation in the model, with priors for larger or more differences doing so more strongly. The exception was Model 6, with forced extended exploration for 10 timesteps, where differences between conditions were harder to determine visually.

A factorial ANOVA using lm() and anova() functions in R 4.0 [37] on final segregation, with model type, priors type, threshold level and their interaction terms, showed a significant 3-way interaction (*F*_40, 8910_ = 418.88, *p* < 0.0001), and when we compared the different patterns of priors in linear models within model type/threshold level combinations, priors for large differences did not result in more segregation than a belief in no differences for Model 6 but belief in small differences (few for 75% threshold or many for all thresholds) continued to lead to slight excess segregation versus an egalitarian prior.

Comparing models within prior type, we see that compared to Model 3 (no real differences), Model 1 always results in stronger segregation regardless of prior type, while Models 5 and 6 (forced exploration) results in less segregation in all prior types. Model 2 results were very close to Model 3, and only consistently significantly different in the linear models for 90% and 100% thresholds. Model 4 (initial differences that disappear with no forced learning) did not differ at the final timestep from Model 3.

We also observed that more inertia changing roles, (agents tolerating a lower threshold before switching roles) exaggerates the effects of priors, since agents are more likely to remain in a role that they chose early in their life based heavily on social information. A 75% inertia threshold leads to very high levels of segregation, especially with priors for many differences.

Importantly, when we compare to the segregation observed among agents with ‘perfect knowledge’ in figure 2, we see that priors for group differences bring agents closer to that level of segregation in Models 1 and 2, except for when the threshold drops too low, and the levels of segregation begin to overshoot ‘optimal’ segregation outcomes. In Models 3–6, where no real differences exist (either right from the start, or from later on), the ‘optimal’ segregation is equal to the egalitarian priors condition, and priors for group differences always lead to ‘excessive’ segregation compared to optimal distribution.

Our key finding is thus that while own-group copying may lead to ‘appropriate’ segregation where real differences exist between groups in their capacity to perform certain roles, the expectation of group differences and use of social information can induce segregation even in the absence of any such real constraints. Where a population began with strong constraints on roles, which were then removed, role segregation adjusted back to the ‘no constraints’ outcome for the relevant set of priors, likely because of a steady input of naive agents who based their decisions only on knowledge of the current agent population, while all agents lose information regarding their deceased peers.

## 4. Discussion

Here we show that a simple social learning strategy—namely, a tendency to copy self-similar others—can produce a socially segregated labour force irrespective of whether individuals actually differ by type in their ability to perform roles. This pattern persisted when transfers between roles were implausibly easy (i.e. the 100% threshold conditions) and only the extreme measure of forcing agents to spend half their ‘lives’ in individual learning through rotating roles (sometimes) eliminated segregation. In short, segregation was ‘sticky’ whenever copying biases existed.

Our results therefore support social learning biases as at least one potential route to understanding persistent (and often arbitrary) gender differentiation in occupational roles [1], subject choice [38] or personality [23], in societies which have ostensibly reduced/mitigated the social/physiological constraints imposed according to gender. While external enforcement and cognitively complex processes such as stereotyping have a role to play, our model suggests that individuals also self-select into arbitrarily gendered roles as a result of simple social learning biases which, in turn, might produce adaptively gendered behaviour in small-scale societies where physiological sex differences and subsistence patterns may require sharper divisions of labour.

Social learning strategies are argued to be adaptive, even if they sometimes produce maladaptive or arbitrary outcomes, as long as they tend to outperform trial and error learning [7]. Gendered social learning can produce adaptive outcomes in small-scale societies: humans rely on effective and often gendered cooperation in diverse ecological niches [5,39]. Subsistence and forager populations are diverse in their subsistence practices and levels of parental investment, and social learning offers a means for humans to acquire flexible gender roles across ecological niches. Industrialized societies, however, represent an evolutionarily novel landscape in which new forms of social information are abundant. In Model 4 above, we create conditions in which gendered social learning ceases to be a shortcut to finding an appropriate role, and show that even a slight bias towards same-gender copying causes gender segregation in roles to ‘stick’ in the absence of actual gender differences in ability.

Using a social learning lens for understanding the gendering of behaviour, however, has particular power given the flexibility, and the breadth, that such an approach provides. For instance, the fact that children acquire gender attitudes in an arbitrary but also rigidly social way reflects the literature on ‘overimitation’—the copying of entire action sequences even when some parts are clearly functionless. Of particular relevance for understanding gender, children spontaneously enforce the performance of normatively labelled actions in others [40] and they readily attribute normativity to adults' actions without instruction to do so; i.e. they engage in the over-imitation of norms [41]. This demonstrates a clear route to acquisition of clustered socially learned behaviours, whether those behaviours are useful (i.e. representing ‘real differences’) or not.

Socially learned behaviours also serve as a means of solidifying group membership or identity; for instance over-imitation serves a social function and it is more likely in social settings [42]. This offers a potential explanation for recent results from Vishkin *et al.* [43] that parents give their children more distinctively gendered names where or when gender equality indices are higher. They explain this through ‘optimal distinctiveness theory’ and argue that humans may seek to exaggerate markers of group membership where groups are otherwise less distinctive. Where gender is a salient social category, attempts to maintain ‘optimal distinctiveness' might produce the *appearance* of sex differences independent of any actual underlying sex difference in predisposition/preference. Thus, for instance, children may pursue gendered interests and activities which can shape longer-term choices, such as choice of occupation. However, from a social learning perspective, just as increases in the diversity of material culture may increase opportunities for gendered learning, so too do they increase opportunities to acquire or express ‘optimally distinct’ group identity markers.

An additional strength as regards the flexibility of our approach is that although we have modelled a two-type population as a means of understanding gendering of occupations in societies with a strong gender binary, our simulations can be used to model any aspect of identity or self where humans tend to observe and internalize social information from others who are self-similar in some way [14]. This would include, but is not limited to, the formation of gender roles in cultures with more than two gender categories (something that existing psychological models of gender do not explicitly encompass) [3,5].

We do not claim to have ‘solved’ gender role acquisition, but we believe we have demonstrated that there are reasons to expect social learning from self-similar others to be widespread in humans and other species. We have also shown how very simple social learning biases can lead to (partial and arbitrary) gender segregation in social roles, and that the harder it is to move between roles, the more impact these biases have. One key implication from our models, therefore, is that one way to minimize unnecessary gender segregation in occupations is to eliminate the costs of retraining or moving between roles, so that people can more easily find work that suits their skills best. This may include, for instance, starting workplace protections from day one, and a robust social safety net, as well as easy access to adult education. Similarly, forcing extreme levels of individual learning in Model 6 also largely eliminated segregation; we do not advocate steps this dramatic in real life. However, related approaches are being trialled in projects which seek, for instance, to expose girls to STEM activities without gender-role biases being salient (for instance, in all-girls clubs). Although these approaches may be limited by e.g. broader cultural information to which participants have access, policy changes are already underway [44–46] and could potentially expand further, to include providing boys with opportunities to experience female-biased roles.

In our first four models, occupational gender segregation was never eliminated (reduced to the levels of ‘perfect knowledge’ agents) as long as beliefs in sex differences in ability persisted within the population. This raises another key implication: in seeking to understand sex and gender, many psychologists may indirectly be changing what they observe [47]. That is: when publishing results about sex differences and extant gender segregation in personality, social roles or subject choices (publications which are typically widely discussed in the media), the salience of small differences may be increased, increasing expectations of sex differences and therefore exaggerating gender role differences. Many authors in this area are at pains to emphasize their interest in understanding underlying patterns rather than driving a particular position, but they may wish to consider to what extent it is possible for them to avoid becoming a part of the causal pathway they wish to study. See, for instance, DeCasian *et al.* [48] for a discussion of what ‘anti-sexist’ science would be.

We did not find evidence that early constraints on gendered behaviour were entirely maintained through social learning when those constraints disappeared. This is likely because our gradual influx of naive agents had access only to role distribution among still-living agents, so long-term historical patterns had little impact on social learning. By contrast, humans through various routes (storytelling, material culture, visual media) perpetuate long-term retention of previous social information, which may explain those long-term effects (for instance of plough- versus non-plough-subsistence on gender roles) [30]. There is thus a clear need to extend our modelling to encompass this more complex informational input.

Furthermore, our model is simple and focuses only on the roles that actual agents in the population adopt. However, children (and adults) do not form their understanding of gender from only those individuals around them; they also draw on material culture and visual media. Visual media is highly gendered and offers a much wider range of social learning sources than merely the individuals in one's social group [22]. As such, high income capitalist economies—which also tend to have high gender equality indices—likely have a greater degree of potential social learning through these sources (although we note that media connectivity is rapidly expanding across populations who previously had minimal media access) [49]. And even if those media figures could be considered part of the population in the same manner as known individuals, in real human psychology high profile and prestigious individuals may exert unequal influence on the attention and thus social learning of others.

A final caveat to consider is that our model also does not incorporate individual differences. In addition to the fact that priors themselves are likely to vary between individuals, there are known and sometimes systematic individual differences in the propensity to use social learning and these might be important for understanding gender development more broadly. For example, when comparing social learning tendency in a gendered response (potential partner choice) versus non-gendered responses (selecting paintings or hands) there is almost zero variation within individuals, and participants vary between each other in a similar way across all social learning tasks [50]. We can therefore predict that those lower in their reliance on social learning should be less likely to adhere to normative patterns of gender expression. There is interest building in the literature in the association between autistic traits and diverse and/or non-conforming forms of gender expression [51]. We hypothesize that this association is a specific manifestation of a more general pattern: that those who show less conformity to general social roles also do not conform to gendered expectations.

To conclude, we have shown here in a series of agent-based models that very simple learning biases may cause individuals to self-socialize and self-segregate into both ‘adaptive’ and arbitrary gender roles within a population. More gender-equal countries are typically wealthier and with a greater range of both sources of cultural information (via media) and potential cultural niches, which likely magnify the effects of social learning. We therefore argue that so-called ‘modern’ and more gender-equal societies do not ‘release’ our evolved psychology: rather, they present a barrage of evolutionarily novel information which we cannot and must not exclude from our understanding of the evolution of sex and gender.

## Data Availability

Data and relevant code for this research work are stored in GitHub: https://github.com/ChrisJefferson/sexdiffpub and have been archived within the Zenodo repository: https://zenodo.org/record/8017254 [35]. Additional information is provided in electronic supplementary material [52].

## References

[1] Anker R. 1998 Gender and jobs: Sex segregation of occupations in the world. Geneva: International Labour Office.

[2] Stoet G, Geary DC. 2018 The gender-equality paradox in science, technology, engineering, and mathematics education. Psychol. Sci. **29**, 581-593. (10.1177/0956797617741719) 29442575

[3] Schmitt DP. 2015 The Evolution of Culturally-Variable Sex Differences: Men and Women Are Not Always Different, but When They Are…It Appears Not to Result from Patriarchy or Sex Role Socialization. In The evolution of sexuality (eds TK Shackelford, RD Hansen), pp. 221-256. Berlin, Germany: Springer International Publishing.

[4] Giolla M, & Kajonius E, J P. 2019 Sex differences in personality are larger in gender equal countries: Replicating and extending a surprising finding. Int. J. Psychol. **54**, 705-711. (10.1002/ijop.12529) 30206941

[5] Wood W, Eagly AH. 2012 Biosocial Construction of Sex Differences and Similarities in Behavior. In Advances in experimental social psychology, pp. 55-123: Elsevier.

[6] Hoppitt W, Laland KN. 2013 Social learning: an introduction to mechanisms, methods, and models. Princeton: Princeton University Press.

[7] Morgan TJH, Rendell LE, Ehn M, Hoppitt W, Laland KN. 2012 The evolutionary basis of human social learning. Proc. R. Soc. B **279**, 653-662. (10.1098/rspb.2011.1172) PMC3248730 21795267

[8] Arbilly M, Motro U, Feldman MW, Lotem A. 2011 Evolution of social learning when high expected payoffs are associated with high risk of failure. J. R. Soc. Interface **8**, 1604-1615. (10.1098/rsif.2011.0138) 21508013 PMC3177617

[9] Kendal R, Hopper LM, Whiten A, Brosnan SF, Lambeth SP, Schapiro SJ, Hoppitt W. 2015 Chimpanzees copy dominant and knowledgeable individuals: implications for cultural diversity. Evol. Hum. Behav. **36**, 65-72. (10.1016/j.evolhumbehav.2014.09.002) 27053916 PMC4820294

[10] Barrett BJ, McElreath RL, Perry SE. 2017 Pay-off-biased social learning underlies the diffusion of novel extractive foraging traditions in a wild primate. Proc. R. Soc. B **284**, 20170358. (10.1098/rspb.2017.0358) PMC5474070 28592681

[11] Rendell L, Fogarty L, Hoppitt WJ, Morgan TJ, Webster MM, Laland KN. 2011 Cognitive culture: theoretical and empirical insights into social learning strategies. Trends Cogn. Sci. **15**, 68-76. (10.1016/j.tics.2010.12.002) 21215677

[12] Kendal RL, Boogert NJ, Rendell L, Laland KN, Webster M, Jones PL. 2018 Social learning strategies: bridge-building between fields. Trends Cogn. Sci. **22**, 651-665. (10.1016/j.tics.2018.04.003) 29759889

[13] Morgan TJH, Laland KN, Harris PL. 2015 The development of adaptive conformity in young children: effects of uncertainty and consensus. Dev. Sci. **18**, 511-524. (10.1111/desc.12231) 25283881

[14] Wood LA, Kendal RL, Flynn EG. 2013 Whom do children copy? Model-based biases in social learning. Dev. Rev. **33**, 341-356. (10.1016/j.dr.2013.08.002)

[15] Eble A, Hu F. 2022 Gendered beliefs about mathematics ability transmit across generations through children's peers. Nat. Hum. Behav. **6**, 868-879. (10.1038/s41562-022-01331-9) 35393520

[16] Pasterski V, Zucker KJ, Hindmarsh PC, Hughes IA, Acerini C, Spencer D, Neufeld S, Hines M. 2015 Increased cross-gender identification independent of gender role behavior in girls with congenital adrenal hyperplasia: results from a standardized assessment of 4- to 11-year-old children. Arch. Sex. Behav. **44**, 1363-1375. (10.1007/s10508-014-0385-0) 25239661

[17] Liberman Z, Woodward AL, Kinzler KD. 2017 The origins of social categorization. Trends Cogn. Sci. **21**, 556-568. (10.1016/j.tics.2017.04.004) 28499741 PMC5605918

[18] Martin CL, Ruble D. 2004 Children's search for gender cues: cognitive perspectives on gender development. Curr. Dir. Psychol. Sci. **13**, 67-70. (10.1111/j.0963-7214.2004.00276.x)

[19] Perry DG, Bussey K. 1979 The social learning theory of sex differences: imitation is alive and well. J. Pers. Soc. Psychol. **37**, 1699-1712. (10.1037/0022-3514.37.10.1699)

[20] Hines M, Pasterski V, Spencer D, Neufeld S, Patalay P, Hindmarsh PC, Hughes IA, Acerini CL. 2016 Prenatal androgen exposure alters girls' responses to information indicating gender-appropriate behaviour. Phil. Trans. R. Soc. B **371**, 20150125. (10.1098/rstb.2015.0125) 26833843 PMC4785908

[21] Fine C, Dupré J, Joel D. 2017 Sex-linked behavior: evolution, stability, and variability. Trends Cogn. Sci **21**, 666-673. (10.1016/j.tics.2017.06.012) 28821346

[22] Boothroyd L, Cross C. 2022 (Super-)cultural clustering explains gender differences too. Behavioural Brain Sciences **45**, e156. (10.1017/S0140525X21001539) 36098408

[23] Smaldino PE, Lukaszewski A, von Rueden C, Gurven M. 2019 Niche diversity can explain cross-cultural differences in personality structure. Nat. Hum. Behav. **3**, 1276-1283. (10.1038/s41562-019-0730-3) 31527682

[24] Durkee PK, Lukaszewski AW, von Rueden CR, Gurven MD, Buss DM, Tucker-Drob EM. 2022 Niche diversity predicts personality structure across 115 nations. Psychol. Sci. **33**, 285-298. (10.1177/09567976211031571) 35044268 PMC13171031

[25] Abbate J. 2017 Recoding gender: women's changing participation in computing. Cambridge, MA: MIT Press Ltd.

[26] Lew-Levy S et al. 2022 Socioecology shapes child and adolescent time allocation in twelve hunter-gatherer and mixed-subsistence forager societies. Sci. Rep. **12**, 8054. 35577896 10.1038/s41598-022-12217-1 PMC9110336

[27] Vashro L, Padilla L, Cashdan E. 2016 Sex differences in mobility and spatial cognition: a test of the fertility and parental care hypothesis in northwestern namibia. Hum. Nat. **27**, 16-34. (10.1007/s12110-015-9247-2) 26577341

[28] Trumble BC, Gaulin SJC, Dunbar MD, Kaplan H, Gurven M. 2016 No sex or age difference in dead-reckoning ability among tsimane forager-horticulturalists. Hum. Nat. **27**, 51-67. (10.1007/s12110-015-9246-3) 26590826

[29] Spence I, Feng J. 2010 Video games and spatial cognition. Rev. Gen. Psychol **14**, 92-104. (10.1037/a0019491)

[30] Alesina A, Giuliano P, Nunn N. 2011 Fertility and the plough. Am. Econ. Rev **101**, 499-503. (10.1257/aer.101.3.499)

[31] Krems JA, Claessens S, Fales MR, Campenni M, Haselton MG, Aktipis A. 2021 An agent-based model of the female rivalry hypothesis for concealed ovulation in humans. Nat. Hum. Behav. **5**, 726-735. (10.1038/s41562-020-01038-9) 33495572

[32] Archer J. 2013 Human sex differences in aggression from the perspective of sexual selection. In Aggression in humans and primates: biology, psychology, sociology (eds J Heinze, H Kortüm), pp. 101-119.

[33] Hines M, Spencer D, Kung KT, Browne WV, Constantinescu M, Noorderhaven RM. 2016 The early postnatal period, mini-puberty, provides a window on the role of testosterone in human neurobehavioural development. Curr. Opin. Neurobiol. **38**, 69-73. (10.1016/j.conb.2016.02.008) 26972372

[34] Hyde JS. 2014 Gender similarities and differences. Annu. Rev. Psychol. **65**, 373-398. (10.1146/annurev-psych-010213-115057) 23808917

[35] Cross CP, Boothroyd LG, Jefferson CA. 2023 Code for: Agent-based models of the cultural evolution of occupational gender roles. Zenodo. (10.5281/zenodo.8017254) PMC10300665 37388313

[36] Wickham H. 2016 ggplot2: elegant graphics for data analysis. New York: Springer.

[37] R Core Team. 2020 R: A Language and Environment for Statistical Computing.

[38] Tolsma J, Need A, de Jong U. 2010 Explaining participation differentials in dutch higher education: the impact of subjective success probabilities on level choice and field choice. Eur. Sociol. Rev. **26**, 235-252. (10.1093/esr/jcp061)

[39] Sear R. 2016 Beyond the nuclear family: an evolutionary perspective on parenting. Curr. Opin. Psychol. **7**, 98-103. (10.1016/j.copsyc.2015.08.013)

[40] Hoehl S, Keupp S, Schleihauf H, McGuigan N, Buttelmann D, Whiten A. 2019 ‘Over-imitation’: a review and appraisal of a decade of research. Dev. Rev. **51**, 90-108. (10.1016/j.dr.2018.12.002)

[41] Schmidt MFH, Rakoczy H, Tomasello M. 2011 Young children attribute normativity to novel actions without pedagogy or normative language: young children attribute normativity. Dev. Sci. **14**, 530-539. (10.1111/j.1467-7687.2010.01000.x) 21477192

[42] Over H, Carpenter M. 2012 Putting the social into social learning: explaining both selectivity and fidelity in children's copying behavior. J. Comp. Psychol. **126**, 182-192. (10.1037/a0024555) 21767011

[43] Vishkin A, Slepian ML, Galinsky AD. 2022 The gender-equality paradox and optimal distinctiveness: more gender-equal societies have more gendered names. Soc. Psychol. Personal. Sci. **13**, 490-499. (10.1177/19485506211037576)

[44] Dasgupta N, Stout JG. 2014 Girls and women in science, technology, engineering, and mathematics: STEMing the Tide and Broadening Participation in STEM Careers. Policy Insights Behav. Brain Sci. **1**, 21-29. (10.1177/2372732214549471)

[45] United Nations Children's Fund, ITU. 2020 *Towards an equal future: Reimagining girls' education through STEM*. See https://www.unicef.org/media/84046/file/Reimagining-girls-education-through-stem-2020.pdf

[46] Department for Education, Behavioural Insights Team. 2020 *Applying Behavioural Insights to increase female students’ uptake of STEM subjects at A Lev*. See https://assets.publishing.service.gov.uk/government/uploads/system/uploads/attachment_data/file/938848/Applying_Behavioural_Insights_to_increase_female_students__uptake_of_STEM_subjects_at_A_Level.pdf

[47] Schudson ZC. 2021 Psychology's Stewardship of Gender/Sex. Perspect. Psychol. Sci. **16**, 1105-1112. (10.1177/17456916211018462) 34726098

[48] DeCasien AR, Guma E, Liu S, Raznahan A. 2022 Sex differences in the human brain: a roadmap for more careful analysis and interpretation of a biological reality. Biol. Sex Differ. **13**, 43. (10.1186/s13293-022-00448-w) 35883159 PMC9327177

[49] Boothroyd LG, Jucker JL, Thornborrow T, Barton RA, Burt DM, Evans EH, Jamieson MA, Tovée MJ. 2020 Television consumption drives perceptions of female body attractiveness in a population undergoing technological transition. J. Pers. Soc. Psychol. **119**, 839-860. (10.1037/pspi0000224) 31854999

[50] Street SE, Morgan TJ, Thornton A, Brown GR, Laland KN, Cross CP. 2018 Human mate-choice copying is domain-general social learning. Sci. Rep. **8**, 1715. (10.1038/s41598-018-19770-8) PMC5788917 29379046

[51] Corbett BA, Muscatello RA, Klemencic ME, West M, Kim A, Strang JF. 2023 Greater gender diversity among autistic children by self-report and parent-report. Autism **27**, 158-172. (10.1177/13623613221085337) 35363085 PMC9525458

[52] Cross CP, Boothroyd LG, Jefferson CA. 2023 Agent-based models of the cultural evolution of occupational gender roles. Figshare. (10.6084/m9.figshare.c.6700187) PMC10300665 37388313

